# Does Coral Disease Affect *Symbiodinium*? Investigating the Impacts of Growth Anomaly on Symbiont Photophysiology

**DOI:** 10.1371/journal.pone.0072466

**Published:** 2013-08-14

**Authors:** John Henrik Robert Burns, Toni Makani Gregg, Misaki Takabayashi

**Affiliations:** 1 Tropical Conservation Biology and Environmental Science, University of Hawai‘i at Hilo, Hilo, Hawai’i, United States of America; 2 Marine Science Department, University of Hawai‘i at Hilo, Hilo, Hawai’i, United States of America; University of Sydney, Australia

## Abstract

Growth anomaly (GA) is a commonly observed coral disease that impairs biological functions of the affected tissue. GA is prevalent at Wai ‘ōpae tide pools, southeast Hawai ‘i Island. Here two distinct forms of this disease, Type A and Type B, affect the coral, *Montipora* *capitata* . While the effects of GA on biology and ecology of the coral host are beginning to be understood, the impact of this disease on the photophysiology of the dinoflagellate symbiont, *Symbiodinium* spp., has not been investigated. The GA clearly alters coral tissue structure and skeletal morphology and density. These tissue and skeletal changes are likely to modify not only the light micro-environment of the coral tissue, which has a direct impact on the photosynthetic potential of *Symbiodinium* spp., but also the physiological interactions within the symbiosis. This study utilized Pulse amplitude modulation fluorometry (PAM) to characterize the photophysiology of healthy and GA-affected *M* *. capitata* tissue. Overall, endosymbionts within GA-affected tissue exhibit reduced photochemical efficiency. Values of both F_v_/F_m_ and ΔF/ F_m_’ were significantly lower (p<0.01) in GA tissue compared to healthy and unaffected tissues. Tracking the photophysiology of symbionts over a diurnal time period enabled a comparison of symbiont responses to photosynthetically available radiation (PAR) among tissue conditions. Symbionts within GA tissue exhibited the lowest values of ΔF/F_m_’ as well as the highest pressure over photosystem II (p<0.01). This study provides evidence that the symbionts within GA-affected tissue are photochemically compromised compared to those residing in healthy tissue.

## Introduction

Reports of diseases affecting marine organisms have increased steadily over the last several years [1]. Scleractinian corals, in particular, have suffered from large-scale disease outbreaks resulting in mortality and phase shifts from coral to algal dominated reefs [1–4]. Reported increases in coral disease prevalence is likely driven by factors such as thermal stress, eutrophication, and disturbance events [5–8]. Indo-Pacific coral reefs, despite being less prone to detrimental disease outbreaks than other regions around the globe, have experienced a proliferation of coral diseases [4,9–12]. While some coral diseases have been extensively studied, factors such as etiology, transmissibility, pathogenesis, and mortality for many diseases remain largely uncharacterized [4,13–15].

Corals exist in a dynamic equilibrium with a diverse community of microorganisms including bacteria, viruses, fungi, endolithic algae, and *Symbiodinium* [16–18]. The mutualistic symbiosis between hermatypic scleractinian corals and photosynthetic endosymbiotic dinoflagellates in the genus *Symbiodinium* is essential for coral health and productivity [19–21]. Biotic and abiotic stressors may induce physiological disruptions to this relationship resulting in compromised immunity of the coral host and manifestation of disease signs [18,22–24]. Considering the importance of *Symbiodinium* to the health and function of the coral holobiont, it is critical to investigate the impacts of emerging diseases on *Symbiodinium* photophysiology.

*In vivo* chlorophyll *a* fluorescence techniques have been successfully utilized to investigate the photophysiological responses of *Symbiodinium* to a variety of stressors [18,25,26]. Pulse amplitude modulation (PAM) fluorometry allows for determination of the ratio of variable chlorophyll fluorescence (F_v_) to maximum fluorescence (F_m_) within the symbiotic dinoflagellate. The F_v_/F_m_ ratio is indicative of the efficiency of photosystem II (PSII) charge separation and enables comparisons of photophysiological performance [27–29]. F_v_/F_m_ values are dependent on the photophysiological function of symbiotic dinoflagellates, not simply symbiont or pigment density, thus decreases in F_v_/F_m_ imply a level of stress imposed on photochemical efficiency [25,29–31].

Growth anomaly (GA) is a prominent coral disease that has been reported from reefs throughout the world [1,32,33]. Among scleractinians, the acroporids appear to be the family of corals most affected by this disease. The gross morphology of GA is characterized by protuberant skeletal growth, pale appearance, and loss of polyp structure [32–36]. Histological studies investigating the uncoordinated growth found in GA has identified hyperplasia of the basal body wall, as well as a reduction in cellular components, to be intrinsic characteristics of this disease [10,12,32,35,36]. The observed reduction in polyps, mesenterial filaments, nematocytes, and gonads has elucidated that this disease affects critical biological functions such as reproduction, feeding, digestion, defense, and energy acquisition [12,34,35]. Biochemical studies have reported depletion of storage lipids within GA-affected tissues, thus suggesting this disease causes an increased energy demand for sustained tissue growth [37]. To date, no microbial studies have successfully identified any specific infectious agent associated with GA, and only one study has experimentally demonstrated transmission of this disease between colonies [32–36,38].

**Figure 1:**
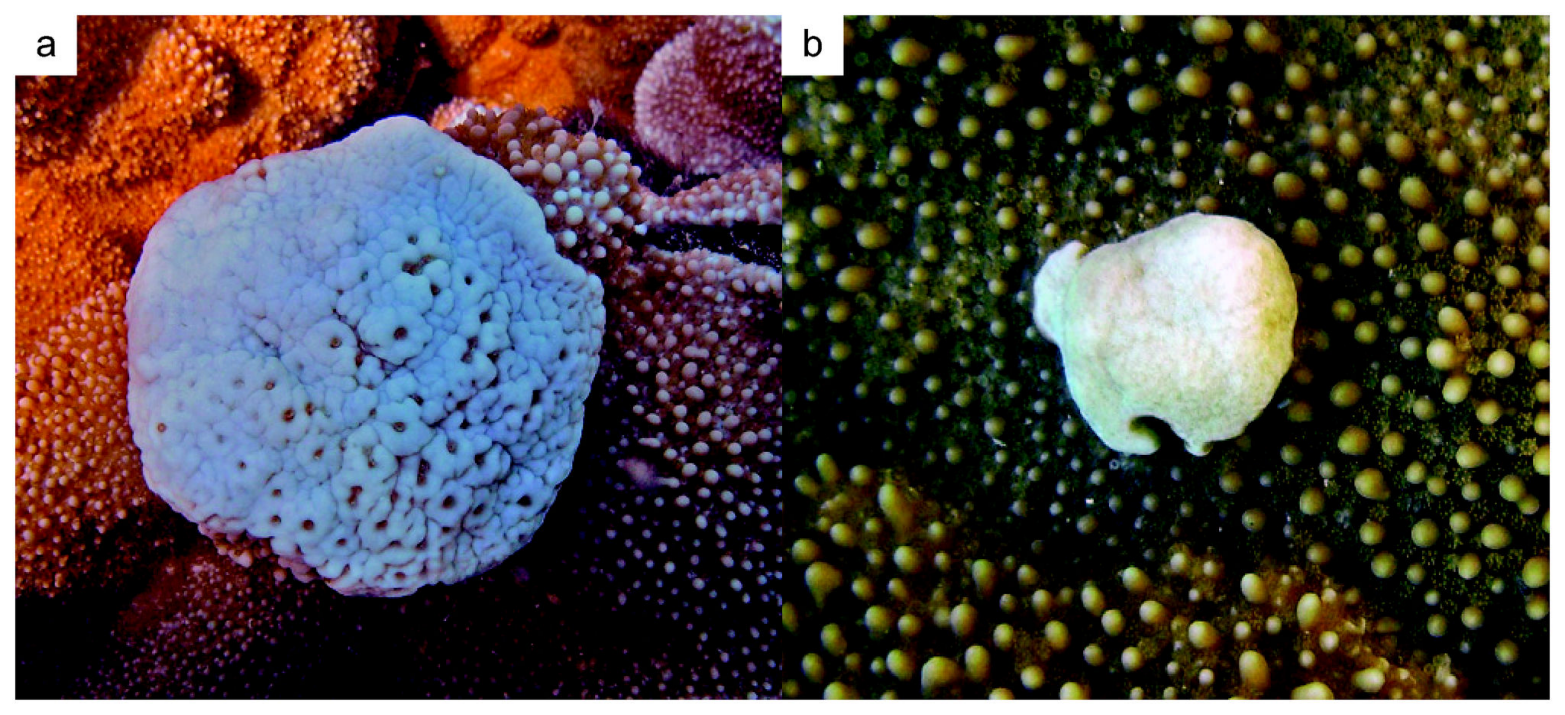
Type A and Type B GA morphology. Photographs of Type A and Type B GA tissue. **a** Type A GA, note reduction in polyps, fused tuberculae, and pale appearance **b** Type B GA, note complete lack of polyps, fused protuberant coenosteum, and pale appearance.

The coral, *Montipora* *capitata* , inhabiting the Wai‘ōpae tide pools along southeast Hawai‘i Island exhibits a higher prevalence of GAs than any other surveyed sites throughout the Hawaiian archipelago [39–41]. The GA lesions affecting *M* *. capitata* at this site display the following morphological characteristics; exophytic nodular protrusion, distinct and undulating margins, pale appearance, and fusion of tuberculae [34]. Two distinct morphological forms of this disease affect *M* *. capitata* corals, Type A and Type B (Figure 1), with Type B exhibiting more degenerative features of this disease [34,35]. This study utilized PAM fluorometry to characterize the impacts of Type A and B GAs on *Symbiodinium* photophysiology. Furthermore, the density and genotype of symbionts were analyzed from tissue used for PAM measurements. Investigating *Symbiodinium* characteristics within healthy and diseased *M* *. capitata* tissue enabled quantification of GA impacts to the endosymbiotic component of the *M* *. capitata* coral holobiont.

## Methods

Fluorescence measurements were taken *in situ* using an underwater pulse amplitude modulated fluorometer (Diving-PAM, Walz, Germany) on *M* *. capitata* colonies at Wai‘ōpae tide pools, southeast Hawai‘i Island (19°29’55″ N 154°49’06″ W). Values of F_v_/F_m_ are obtained via PAM fluorometry by measuring initial chlorophyll fluorescence (F_0_) with a weak pulsed light source and then applying saturating white light causing a reduction of PSII reaction centers which increases fluorescence to a maximum value (F_m_). Variable fluorescence (F_v_) is the difference between the values of F_0_ and F_m_ (Jones et al. 2000, Maxwell and Johnson 2000). Coral fragments (<2 cm diameter) were collected from *M* *. capitata* colonies, with permission from the State of Hawai’i Department of Land and Natural Resources, Division of Aquatic Resources, from the areas on each *M* *. captiata* colony used for PAM measurements during the diurnal photophysiology experiment. A portion of each sample was fixed in guanidinium for genetic analysis, and the rest was fixed in a 1:4 zinc-formalin: filtered seawater (2µm) fixative (Z-Fix, Anatech, Ltd.) to analyze the density of *Symbiodinium*.

### Comparison of symbiont photochemical efficiency between healthy and GA tissue

Fluorescence measurements were taken on healthy tissue, unaffected tissue (apparently healthy tissue from a diseased colony), and Type A and B GA lesions to compare photochemical efficiency among these tissue conditions. 36 individual colonies were sampled for the healthy (n=12), Type A (n=12), and Type B (n=12) tissue conditions; unaffected measurements were taken from an apparently healthy part of the colonies affected by Type A and B GAs. An opaque polyvinyl chloride sample holder was used to ensure consistent geometry and distance between the fiber-optic cable and coral tissue. Colonies of similar size and morphology were chosen for analysis. Measurements of unaffected tissues were taken proximal (<10mm) to GA lesions as well as distal (>30cm) from lesions to determine if proximity to GA lesions affects symbiont photophysiology. Measurements of GA tissue were taken from the central area of GA lesions. The saturation width on the Diving-PAM was set to a value of 0.8. The saturation intensity, measuring light intensity, and gain settings were adjusted and optimized, for measurements taken at solar noon and after dusk, in order to ensure the values of F_o_ (background chlorophyll fluorescence) were within the range of 300-500. Measurements were taken at local solar noon on cloudless days to obtain the maximum light-dependent reduction of the effective quantum yield of PSII (ΔF/F_m_’) relative to its maximum value recorded after dusk (F_v_/F_m_). Maximum excitation pressure over PSII was calculated as: Q_m_=1-[(ΔF/F_m_’ at noon)/(F_v_/F_m_ after dusk)] [42]. Irradiance was measured with the photosynthetically active radiation (PAR) sensor of the Diving-PAM previously calibrated against a 2π Li-COR light sensor.

### Diurnal variability in photophysiology between healthy and GA tissue

Fluorescence measurements were taken hourly beginning pre-dawn (6: 00) and continuing until after sunset (19: 00) to track diurnal variability in the photophysiology of healthy, unaffected, and Type A and B GA tissue. 9 individual colonies were sampled for the healthy (n=3), Type A (n=3), and Type B (n=3) tissue conditions; unaffected measurements were taken from an apparently healthy part of the colonies affected by Type A and B GAs. The colonies were analyzed hourly, and all measurements of GAs were taken from the central area of the GA lesions. Weighted markers were used to repeatedly align a clear polyvinyl chloride sample holder in the same position to enable measurements from the same area of coral tissue with consistent geometric alignment. Sampling each tissue condition repeatedly throughout the diurnal light cycle, with consistent geometric alignment, enabled the calculation of non-photochemical quenching (NPQ). NPQ was calculated for each tissue condition using the equation NPQ = [(F_m_-F_m_')/F_m_]. Irradiance was measured with the photosynthetic active radiation (PAR) sensor of the Diving-PAM previously calibrated against a 2π Li-COR light sensor.

### Symbiodinium Density

Coral tissue samples were taken from each analyzed portion of the same 9 colonies used for the diurnal analysis described above. Samples used for symbiont analysis were decalcified using Formical bone decalcifier (Decal Chemical Corp., USA) and homogenized using a glass tissue homogenizer. Densities of symbiotic dinoflagellates in each homogenate were calculated from replicate hemacytometer counts.

### Symbiodinium Genotype

DNA was extracted from the above coral tissue samples using the procedure described by 43, and the chloroplast 23S was amplified in a 100µl PCR reaction with 23S1 (GGC TGT AAC TAT AAC GGT CC) and 23S2 (CCA TCG TAT TGA ACC CAG C) primers using the cycling conditions described by 44. The PCR products were purified using Ultra Clean PCR Clean-up DNA Purification Kit (MOBIO) and, cloned as described in [45]. At least six clones from each sample were sequenced at the Advanced Studies of Genomics, Proteomics, and Bioinformatics facility of the University of Hawaii at Mānoa or the Core Genetics Laboratory at the University of Hawai‘i at Hilo.

### Statistical Analysis

Data were transformed, if necessary, using log and arcsine transformations to meet the assumptions of normality and equal variance for use of parametric statistical tests. Variation in mean values of photophysiological parameters was analyzed among the assessed tissue types using a one-way multivariate analysis of variance (MANOVA). The data were further evaluated using univariate ANOVA and Tukey’s HSD post hoc tests to determine statistical differences (α = 0.05) among the examined tissue types. Linear regression analyses and analysis of covariance (ANCOVA) were used to compare photophysiological responses to PAR irradiance among tissue conditions. All statistical analyses were conducted using Minitab 15 (Minitab Inc., State College PA, USA) statistical software.

## Results

Comparison of symbiont photochemical efficiency between healthy and GA afflicted tissues of *M* *. capitata* colonies at Wai‘ōpae

**Figure 2:**
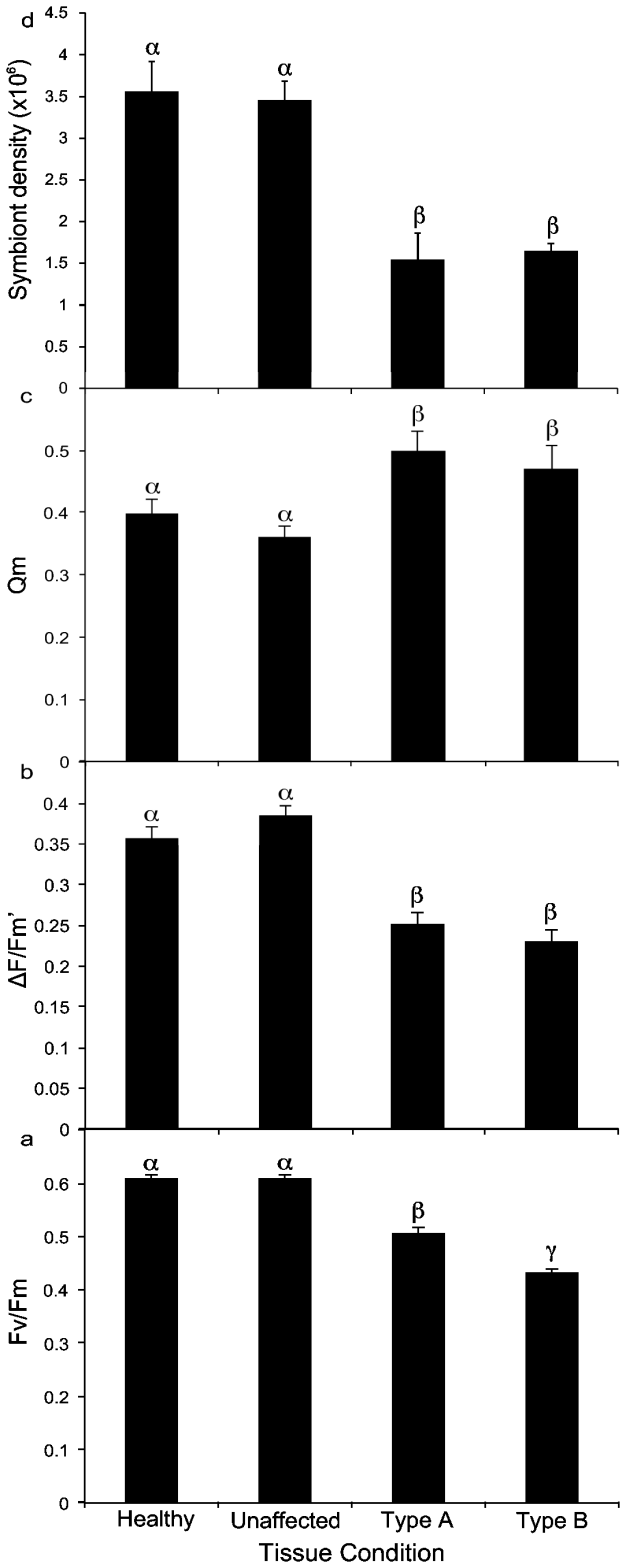
Comparison of photophysiological parameters among tissue conditions. Comparison of photophysiological parameters among healthy, unaffected, Type A GA, and Type B GA tissues of the coral, *Montipora* *capitata* . **a** Mean (± s.e.) values of maximum quantum yield (F_v_/F_m_) **b** Mean (± s.e.) values of effective quantum yield (ΔF/F_m_’) **c** Mean (± s.e.) values of pressure over photosystem II (Q_m_) **d** Mean (± s.e.) symbiont density among the examined tissue conditions in the coral, *Montipora* *capitata* . α, β, and γ denote groupings identified by statistical significance (p<0.01).

Values of ΔF/F_m_’ and F_v_/F_m_ were significantly different among the tissue types (MANOVA, F = 41.41, p<0.001). Further univariate analysis showed Type A and B GAs had significantly lower values of F_v_/F_m_ than healthy and unaffected tissue and F_v_/F_m_ was significantly lower for Type B than Type A GAs (ANOVA, F=145.15, d.f. = 3, p<0.001, Tukey’s HSD p=0.05, Figure 2a). Type A and B GAs also exhibited significantly lower ΔF/F_m_’ values than healthy and unaffected tissue but were not significantly different from each other (ANOVA, F=37.95, d.f. = 3, p<0.001, Tukey’s HSD p=0.05, Figure 2b). Q_m_ in Type A and B GAs were greater than unaffected tissue, but there was no statistical difference between Type A or B GAs (ANOVA, F=37.95, d.f. = 3, p<0.001, Tukey’s HSD p=0.05, Figure 2c).

### Diurnal variability in the photophysiology of healthy and afflicted *M* *. capitata*

**Figure 3:**
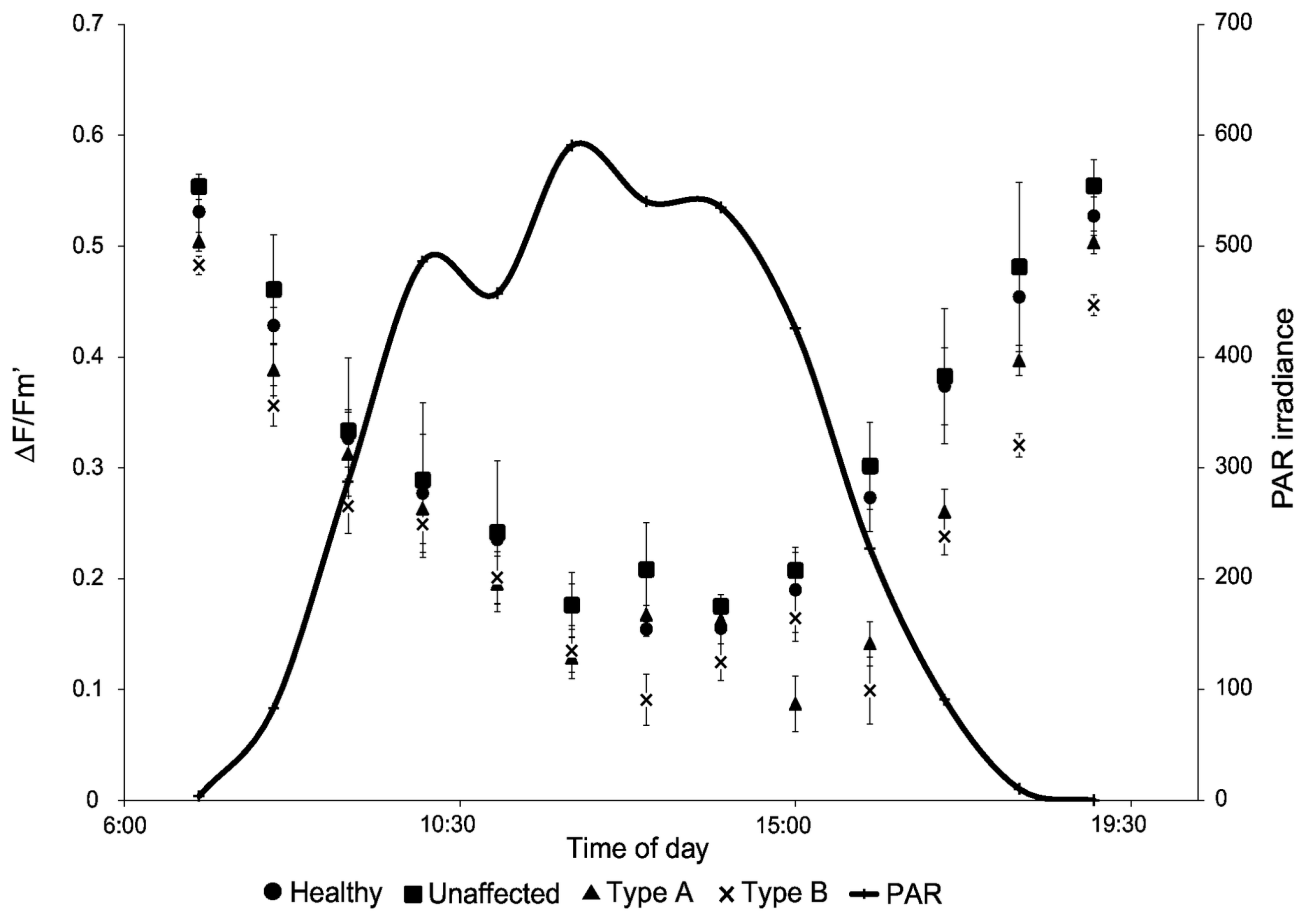
Diurnal tracking of photophysiology among tissue conditions. Values of F_v_/F_m_, ΔF/F_m_’, and PAR obtained during the diurnal experiment.

Type A and B GAs exhibited lower mean values of F_v_/F_m_ and ΔF/F_m_’ than healthy and unaffected tissues throughout the entire period from 06:00 to 19: 00 (Figure 3). Diurnal fluctuations of F_v_/F_m_ and ΔF/F_m_’ in response to photosynthetically available radiation (PAR) were relatively similar among all tissue conditions until noon, at which point ΔF/F_m_’ values from Type A and B tissues did not recover for several hours (Figure 3). Type B was the only tissue condition that did not exhibit complete recovery of F_v_/F_m_ by the end of the experiment.

**Figure 4:**
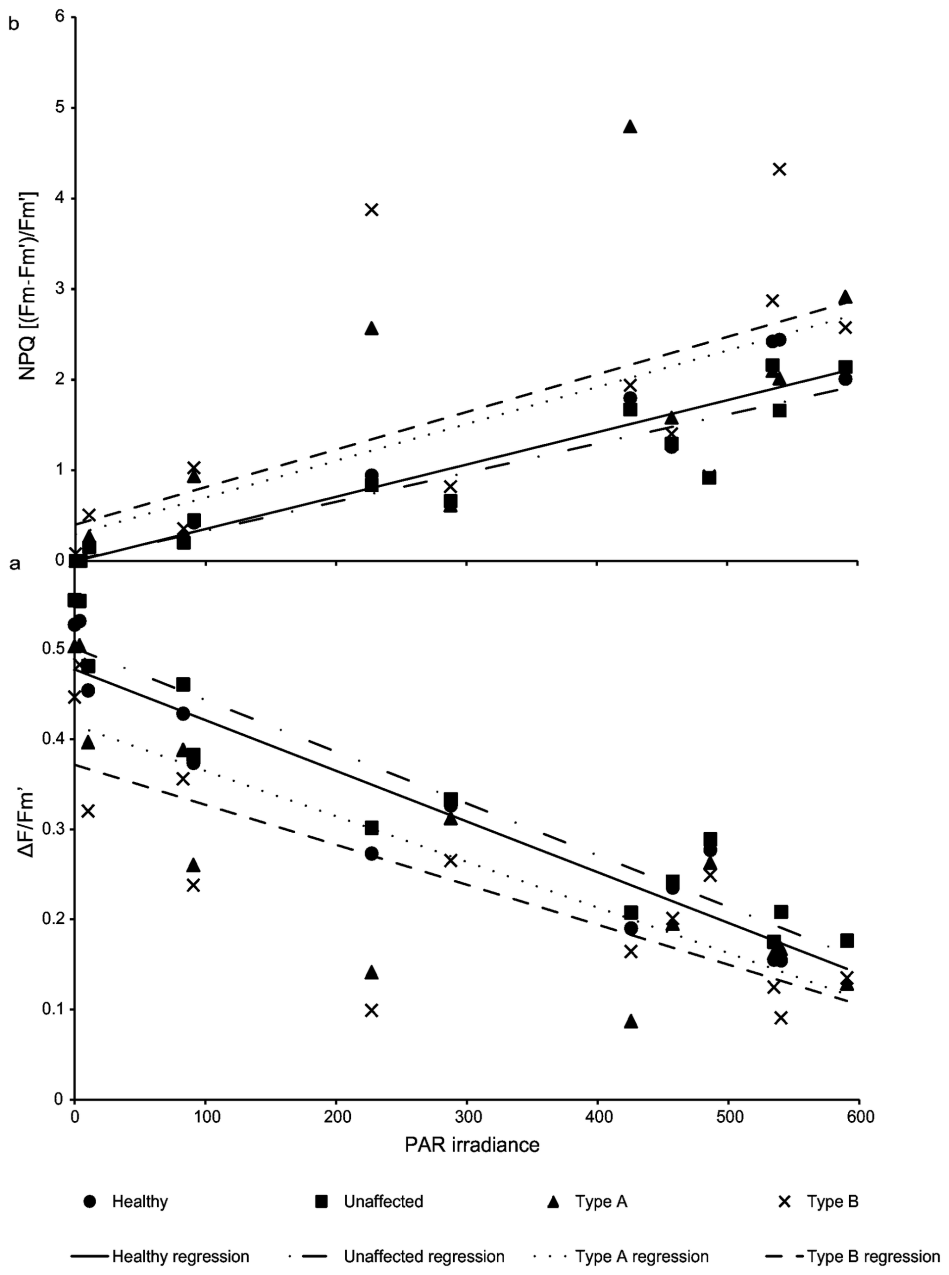
Regression relationships between photophysiological parameters and PAR. **a** Linear regression relationships between ΔF/F_m_’ and PAR for each examined tissue condition **b** Linear regression relationships between NPQ and PAR for each examined tissue condition.

All tissue conditions (healthy, unaffected, Type A and B GAs) exhibited significant relationships between values of ΔF/F_m_’ and PAR (Linear regression, R^2^=63.1-90.0%, F=18.4-98.6, p<0.001, Figure 4a). The slopes of each linear relationship were not significantly different among the examined tissue conditions. Type B GA had a significantly lower y-intercept compared to all other tissue conditions, thus indicating *Symbiodinium* within Type B GA tissue exhibit significantly lower mean values of ΔF/F_m_’ in response to all values of PAR (ANCOVA, R^2^=76.04, F=3.70, p<0.05, Figure 4a).

All tissue types exhibited significant relationships between NPQ and PAR (Linear regression, R^2^=44.0-87.5%, F=8.64-76.96, p<0.05, Figure 4b). The slopes and y-intercepts of each linear relationship did not significantly differ among the examined tissue conditions (Figure 4b).

### Symbiodinium Density

Healthy and unaffected tissue had significantly greater symbiont density than GA tissue; no significant difference was found between healthy and unaffected tissue nor between Type A and Type B GAs (ANOVA, F = 9.67, d.f. = 3, p<0.001, Tukey’s HSD p=0.05, Figure 2d).

### Symbiodinium Genotype

No consistent difference in *Symbiodinium* genetic clades was detected among healthy, unaffected, and GA tissues. Combinations of *Symbiodinium* genotypes found in coral tissues depended on coral colony, rather than the tissue type, and thus values of ΔF/F_m_’ did not correlate with *Symbiodinium* genotypes (Table 1).

**Table 1:** *Symbiodinium* genotype characterization.

			Tissue condition			
	**Healthy**		**Unaffected**		**Growth anomaly**	
**Colony**	Central	Peripheral	Proximal	Distal	Type A	Type B
1	D1	C31, D1	-	-	-	-
2	C31	C31	-	-	-	-
3	C31	C31	-	-	-	-
4	-	-	D1	C31, D1	D1	-
5	-	-	C31	C31, D1, F3	C31, D1	-
6	-	-	C31	C31	C31	-
7	-	-	C31	D1	-	C31
8	-	-	D1	D1	-	D1
9	-	-	D1	D1	-	D1

*Symbiodinium* cp23S genotypes recovered from each examined tissue types (Healthy, Unaffected, Type A GA, and Type B GA) of the coral, *Montipora* *capitata* . All sequences showed a 100% match with sequences on GenBank as: C31 (FN298481); D1(AJ872089); F3 (AJ872095)

## Discussion

The photophysiological performance of *Symbiodinium* is an important measure of coral holobiont metabolic health because the photosynthates translocated from symbionts sustain most, if not all, of the holobiont energetic demands [17,19,20]. Events that compromise the photophysiology of *Symbiodinium*, such as coral bleaching, are often concurrently observed with coral disease outbreaks [8,46,47]. Considering the intimate symbiotic relationship between coral host and algal symbiont, coral disease investigations must address impacts on *Symbiodinium* in order to adequately characterize disease dynamics. Few studies have attempted to analyze photophysiology of symbionts within diseased coral tissue [18,26], and none have attempted to perform *in situ* characterization of symbionts within GA-affected tissue using pulse amplitude modulation fluorometry (PAM).

Growth anomaly (GA) affects many coral species throughout the world’s oceans yet many pathological characteristics of this disease remain poorly understood [12,14,32,36]. The population of the coral, *Montipora* *capitata* , at Wai‘ōpae tide pools on Hawaii Island shows a high prevalence (30.3%) of two distinct morphologic forms of GA, Type A and B (Figure 1), with Type B being the later more detrimental stage of the disease [34,35]. Histopathological analysis has demonstrated this disease causes a reduction in symbiotic dinoflagellate density with associated hyperplasia of the gastrovascular canals [12,32,35,36]. Gross morphological changes induced by the disease, such as fusion of tuberculae and nodular protrusion, may affect *Symbiodinium* photophysiology by altering the micro-shading environment [34,48]. PAM fluorometry provides a useful method for quantifying photophysiological impacts of GA on symbionts within diseased tissue. By comparing maximum (F_v_/F_m_) and effective (ΔF/F_m_’) quantum yield values among tissue conditions, as well as tracking F_v_/F_m_ and ΔF/F_m_’ over diurnal light fluctuations, this study was able to quantify the effects of GA on symbiont photophysiology.

The photochemical efficiency of endosymbionts within GA tissue exhibited significantly reduced values of both F_v_/F_m_ and ΔF/F_m_’ compared to healthy and unaffected tissues (Figure 2a,b). This finding contradicts previous *in vitro* research that found no association between GA-affected tissue and reductions to *Symbiodinium* photochemical efficiency [18,26]. Values of F_v_/F_m_ were lowest in Type B tissue, indicating this form of the disease experiences the greatest loss in photochemical efficiency and/or damage at the level of the PSII reaction centers. This is consistent with prior research indicating Type B GA is the more degenerative stage of this disease [35]. Interestingly, values of ΔF/F_m_’ showed no significant difference between Type A and B GAs (Figure 2b), thus suggesting symbionts within the tissues of both types of GA exhibit similar photochemical efficiency when under maximum light stress. Both Types A and B GA demonstrated heightened levels of excitation pressure over PSII, expressed as Q_m_, compared to healthy and unaffected tissue (Figure 2c). Collectively these findings suggest that symbionts within GA-affected tissue are less photochemically efficient, subjected to a high-light environment, and potentially experiencing higher levels of photoinhibition or down-regulation in PSII photochemistry than symbionts in healthy tissue.

Tracking values of of both F_v_/F_m_ and ΔF/F_m_’ over a diurnal time period from pre-dawn to after sunset (06:00 to 19: 00) enabled determination of fluorescence yields under varying levels of actinic illumination. All tissue conditions displayed a similar declining trend of ΔF/F_m_’ in response to PAR irradiance until solar noon (Figure 3). Interestingly, after solar noon the values of ΔF/F_m_’, for both Type A and Type B, did not begin to recover for several hours. Type A exhibited complete recovery of F_v_/F_m_ by the end of the experiment, while Type B consistently had the lowest values of ΔF/F_m_’ and showed an incomplete recovery of F_v_/F_m_. This post-noon delayed recovery of ΔF/F_m_’ may be occurring due to photoinhibition of *Symbiodinium* within diseased tissue. Further analysis showed that the regression relationships between ΔF/F_m_’ and PAR exhibited similar slopes among all tissue conditions (Figure 4a). The regression y-intercept of Type B tissue was significantly lower than other tissue conditions, thus indicating the photochemical efficiency of *Symbiodinium* within Type B tissue is significantly impaired. Non-photochemical quenching (NPQ) is a photoprotective mechanism in which excess absorbed light energy in the PSII antenna system is dissipated as heat. NPQ values provide an indicator of the *Symbiodinium* photoprotective capability to prevent over-reduction of the photosynthetic electron transport chain. Comparing NPQ values among all tissue conditions determined if *Symbiodinium* within diseased tissue are equally capable of dissipating excess light energy compared to those inhabiting healthy or unaffected tissue. Analysis of NPQ showed all tissue conditions had statistically similar relationships between NPQ and PAR, thus all conditions exhibit a similar capacity for internal photoprotective quenching (Figure 4b). This finding further suggests that higher levels of light stress and photoinhibition are causing the observed reduction of F_v_/F_m_ and ΔF/F_m_’ from *Symbiodinium* within GA-affected tissue.

None of the above differences in photophysiological parameters between GA-affected tissue and healthy tissue is explained by genetic differences of *Symbiodinium* housed by the corals. Though not exhaustive, our sequence analysis of chloroplast 23S suggested that the same *Symbiodinium* genotype combinations were found in unaffected and diseased parts of the same colony (Table 1), yet these different tissue types of the same colony showed different levels of photochemical performance. *Symbiodinium* of clade D has been suggested to dominate in corals under photophysiological stress [49–51]. In our study, however, clade D was recovered from healthy coral as well as colonies hosting Type A and Type B GAs. Since our genotyping method was not quantitative, our data are not indicative of relative proportions *Symbiodinium* clades. However, given the lack of clear differences in cladal composition of *Symbiodinium* between healthy and GA tissues, it can be concluded that it is not the genetic differences in *Symbiodinium* communities that are causing the observed photophysiological differences.

An important question remains; does the disease directly impact the coral host and the symbiont, or is symbiont photophysiology indirectly affected by physical and cellular changes occurring within GA tissue? It is possible that morphological changes, such as fusion of tuberculae and hyperplasia of the gastrovascular cavity [34,35], have altered the light field and the endosymbionts are exposed to higher local irradiances, thus imposing an increased level of light stress on the *Symbiodinium* [35]. The observed reduction in symbiont density within GA tissue (Figure 2d) will further exacerbate light stress by causing an increase in solar reflectance by the coral skeleton as well as reducing the effects of pigment self-shading [52–54]. Studies have shown light reflectance to be intimately dependent on the surface density of *Symbiodinium* cells, and reflectivity increases exponentially as cell density is reduced [54]. This light-amplification may further increase the stress on remaining symbionts causing a positive feedback-loop that accelerates bleaching [55], thus giving GAs their characteristic pale appearance. Considering the molecular identity of the symbionts were not associated with photophysiology, and previous histopathology of GA showed no clear deformation of the symbionts, it is likely that the reduction in symbiont density creates a high-light environment within diseased tissue that has a significant impact on symbiont photochemical efficiency. The fundamental question of whether the GA disease itself causes a reduction in symbiont density, or if the reduction is induced by host morphological changes, must be addressed further to fully characterize the direct effects of this disease on *Symbiodinium*. However, it is clear that the coral host is impacted, as there is a significant reduction in *Symbiodinium* density and photochemical efficiency within GA-affected tissue.

The photophysiological impairment of symbiotic dinoflagellates housed in Type A and B GA has serious implications for the diseased tissue. The immune and metabolic functions compromised in GA-affected coral tissues are likely further exacerbated by the reduction in symbiont density and associated impairment of photochemical efficiency. Determining that GA causes reductions in photophysiological capacity further illustrates that GA is indeed a disease [56], hindering biological function of the coral holobiont. It is clear that as the GA disease progresses from Type A to B morphology, the photophysiological capability of the symbiont decreases and the host tissue loses mechanisms for energy acquisition. Coupled with the complete loss of polyps [34], this could be potentially fatal for GA-affected tissue as means of obtaining energy are severely reduced.

Considering that most investigations on coral health to date gauge the threat of diseases based on simple measures such as prevalence, more work is needed to decipher the actual impacts of disease on coral holobiont function and survival. Investigating the epizootiology, histopathology, and photophysiology of GA affecting *M* *. capitata* has provided critical contributions to assessing the threat of this disease to affected colonies [34,35]. Evaluating the impacts of GA on the cellular and ecological competitiveness of corals, as well as investigation of its pathogenicity are needed to further the etiological understanding of this disease.
